# Δ^9^-Tetrahydrocannabinol (THC) enhances lipopolysaccharide-stimulated tissue factor in human monocytes and monocyte-derived microvesicles

**DOI:** 10.1186/s12950-015-0084-1

**Published:** 2015-06-12

**Authors:** Julie C. Williams, Thomas W. Klein, Bruce A. Goldberger, John W. Sleasman, Nigel Mackman, Maureen M. Goodenow

**Affiliations:** Department of Pathology, Immunology and Laboratory Medicine, College of Medicine, University of Florida, 2033 Mowry Road, Gainesville, FL 32610-3663 USA; Department of Molecular Medicine, Morsani College of Medicine, University of South Florida, Tampa, FL USA; Department of Pediatrics, Division of Allergy, Immunology and Rheumatology, School of Medicine, Duke University, Durham, NC USA; Division of Hematology and Oncology, Department of Medicine, McAlister Heart Institute, University of North Carolina, Chapel Hill, NC USA

**Keywords:** Lipopolysaccharide, Marijuana, Monocyte, Tissue factor, THC

## Abstract

**Background:**

Immunomodulatory effects in humans of Δ^9−^Tetrahydrocannabinol (THC), the psychoactive component of marijuana are controversial. Tissue factor (TF), the activator of the extrinsic coagulation cascade, is increased on circulating activated monocytes and is expressed on microvesicles released from activated monocytes during inflammatory conditions, which perpetuate coagulopathies in a number of diseases. In view of the increased medicinal use of marijuana, effects of THC on human monocytes and monocyte-derived microvesicles activated by lipopolysaccharide (LPS) were investigated.

**Findings:**

Peak levels of TF procoagulant activity developed in monocytes or microvesicles 6 h following LPS treatment and were unaltered by THC. After 24 h of LPS stimulation, TF activity declined in control-treated or untreated cells and microvesicles, but persisted with THC treatment. Peak TF protein occurred within 6 h of LPS treatment independent of THC; by 24 h, TF protein declined to almost undetectable levels without THC, but was about 4-fold greater with THC. Steady-state TF mRNA levels were similar up to 2 h in the presence of LPS with or without THC, while 10-fold greater TF mRNA levels persisted over 3–24 h with THC treatment. Activation of MAPK or NF-κB pathways was unaltered by THC treatment and inflammatory cytokine IL-6 levels were unchanged. In contrast, TNF and IL-8 levels were enhanced by 20–50 %.

**Conclusions:**

THC enhances TF expression in activated monocytes resulting in elevated procoagulant activity. Marijuana use could potentiate coagulopathies in individuals with chronic immune activation such as HIV-1 infection or inflammatory bowel disease.

## Introduction

Tissue factor (TF) is a membrane-bound protein that initiates the extrinsic pathway of the coagulation cascade [1]. *In vitro*, the signaling and kinetics of lipopolysaccharide (LPS)-stimulated TF expression on monocytes and microvesicles are well understood. LPS stimulation of monocytes leads to mitogen activated protein kinase (MAPK) and nuclear factor κB (NF-κB) activation resulting in transcription of TF mRNA followed by translation of TF protein [2–4]. LPS stimulation increases steady state levels of TF mRNA and protein expression, however TF is regulated post-transcriptionally and post-translationally, resulting in a peak expression followed by steady decline [4, 5]. TF expression by monocytes or microvesicles in the circulation is minimal under normal physiologic conditions, while circulating monocytes perturbed by infection or inflammation upregulate TF, and subsequently, release TF via microvesicles [6].

Microvesicles are 100–1000 nm membrane blebs that are released in response to stimulation or cell death [7]. Microvesicles transport cellular signals via their cargo, which can include microRNA, RNA, DNA or proteins [7]. Monocyte-derived microvesicles are significant sources of pro-coagulant activity due to expression of TF, as well as phosphatidylserine, a cofactor for the coagulation cascade.

Individuals with HIV-1 infection and Inflammatory Bowel Disease (IBD) or other diseases with elevated plasma LPS have increased TF expression on monocytes and TF^+^ microvesicles [8, 9] and are at increased risk for coagulopathies [8, 10]. Marijuana is proposed for pharmacological interventions for either HIV-1 infection or IBD [11, 12]. The psychoactive component of marijuana, Δ^9−^tetrahydrocannabinol (THC), has immunomodulatory properties, although most studies showing that THC is anti-inflammatory were performed in animal models, murine cells or transformed human cell lines [13]. Human monocytes express THC receptors [13], however the effects of THC on human monocytes, microvesicles, and coagulation are unknown. Here, we investigated the effects of THC on LPS-stimulated TF expression and activity in human monocytes and monocyte-derived microvesicles.

## Methods

### Cells and reagents

Elutriated human monocytes were obtained from Dr. Mark Wallet at the University of Florida under protocols approved by the Institutional Review Board. Monocytes were rested overnight in Dulbecco’s Modified Eagle Medium (DMEM) (Corning) containing 10 % human serum, Ciprofloxacin (Corning), and Gentamicin (Sigma) prior to addition of THC (Sigma) or ethanol vehicle control. In all experiments, THC or vehicle alone was added 30 min prior to stimulation with LPS from E.coli O111:B4 (Sigma).

### Isolation of microvesicles and flow cytometry

Microvesicles were isolated from cell and cellular debris free supernatants by centrifugation at 16,000xg for 15 min at 4 °C and Annexin V FITC staining observed by flow cytometry, as previously described [14].

### Tissue factor activity assay

TF procoagulant activity assay was performed as previously described [15].

### Protein analysis: Western Blot and ELISA

Whole cell lysates were obtained using lysis buffer (Cell Signaling Technology) from monocytes stimulated with THC or vehicle 30 min prior to 100 ng/mL LPS for indicated time periods. Lysates were analyzed by sodium dodecyl sulfate-polyacrylamide gel electrophoresis (SDS-PAGE) and transferred to polyvinyl difluoride (PVDF) membrane (Bio-Rad). Membranes were probed using anti-TF antibody (BD Biosciences) or antibodies to actin, pERK1/2, total ERK1/2, phospho-p65, total p65, or IκBα (Cell Signaling Technology). Densitometry was performed in Image J (NIH). Band intensity ratios of TF to actin were quantified and then normalized to LPS only treatments. ELISA for human interleukin-6 (IL-6), tumor necrosis factor-α (TNFα), interleukin-8 (IL-8) (BD Biosciences) were performed according to manufacturer’s instructions.

### RNA isolation and quantitative real-time PCR

RNA was isolated and reverse transcribed into cDNA as previously described [16]. Quantitative real-time PCR (qPCR) was performed using primers and probes for TF, IL-6, TNFα, IL-8 and gyceraldehyde 3-phosphate dehydrogenase (GAPDH) (IDT Technologies) using an ABI 7500 FAST instrument.

### Statistical analysis

Statistical analysis using ANOVA followed by Bonferoni post test was performed in GraphPad Prism. A *p* value less than 0.05 was considered statistically significant.

## Results and discussion

**Fig. 1:**
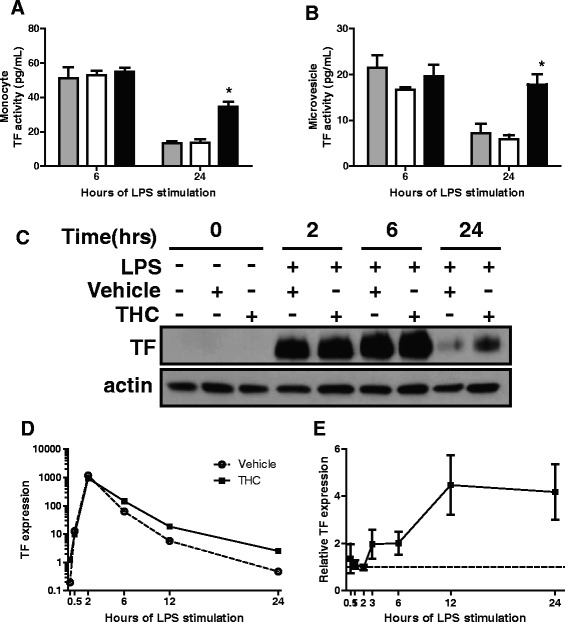
THC prolongs TF expression and procoagulant activity. Monocytes were treated with 30 μM THC or vehicle control for 30 min prior to the addition of LPS (100 ng/mL) for indicated time periods. TF activity in (**a**) monocytes or (**b**) microvesicles isolated from monocyte supernatants in (**a**) was measured. Graphs are results are from one donor, showing mean and standard error from 3 wells. Similar results were obtained in cells from 3 donors. TF activity is donor dependent, and, in the absence of LPS stimulation, usually undetectable, but never greater than 1 pg/mL in media only or THC only controls. Grey bars are LPS only, open bars are vehicle control and LPS, solid black bars are THC and LPS. * *p* < 0.001 via ANOVA followed by Bonferroni post test comparing THC to vehicle. **c** Whole cell lysates from monocytes were analyzed by western blot for indicated proteins. TF western blots were stripped and reprobed for actin. **d**, **e** Total mRNA was isolated from monocytes treated with THC or vehicle 30 min prior to LPS and analyzed for TF and GAPDH by quantitative real-time PCR. **d** TF versus GAPDH mRNA from one representative donor is graphed. Data is presented as a relative fold change compared to untreated cells. **e** Mean and standard error from at least 3 donors are graphed relative to vehicle (dotted line at 1) at indicated time points

The effect of THC pretreatment on LPS-stimulated TF procoagulant activity of monocytes was measured over the course of 24 h. Peak TF activity occurred after 6 h of LPS stimulation independent of THC treatment (Fig. 1a). By 24 h, TF procoagulant activity remained elevated in THC-treated LPS-stimulated monocytes, but declined significantly in vehicle-treated or untreated LPS-stimulated cells (Fig. 1a). Similar to monocyte TF activity, peak microvesicle TF activity occurred by 6 h of LPS stimulation (Fig. 1b). After 24 h, TF activity of microvesicles from THC-treated cells was approximately 3-fold greater compared to vehicle-treated or untreated LPS stimulated MVs (Fig. 1b). Although the magnitude of monocyte and microvesicle TF activity was donor dependent, THC-mediated increased TF activity occurred with a range of 2- to 6-fold at 24 h among donors. Results indicate that THC modulation of TF activity in monocytes was paralleled by elevation in microvesicle TF activity.

To confirm that TF activity was the result of elevated TF protein expression and not dependent on a co-factor or post-translational modification, TF protein levels were evaluated by western blot. TF protein appeared by 2 h post LPS stimulation (Fig. 1c). No differences in levels were apparent until 24 h of LPS stimulation, when THC-treated cells had greater levels of TF protein compared to cells treated with vehicle control. As expected, TF protein was undetectable in the absence of LPS stimulation (data not shown). Next, TF mRNA levels were evaluated over 24 h by qPCR. TF mRNA levels in THC-treated and vehicle control-treated cells remained similar until peak expression 2 h post LPS stimulation, but were elevated in THC-treated cells at all subsequent time points (Fig. 1d). Among 5 donors, levels of TF mRNA in the presence of THC were approximately 2-fold higher at 3 and 6 h, increasing to 4-fold higher by 12 and 24 h (Fig. 1e). Although the magnitude of THC responses differed among donors, all donors showed THC-mediated elevated TF expression from 2 to 5-fold relative to control over 3 to 24 h of LPS stimulation (Fig. 1e). Findings indicate that THC treatment prolongs levels of TF mRNA and protein, as well as TF procoagulant activity, in both monocytes and monocyte-derived microvesicles.

**Fig. 2:**
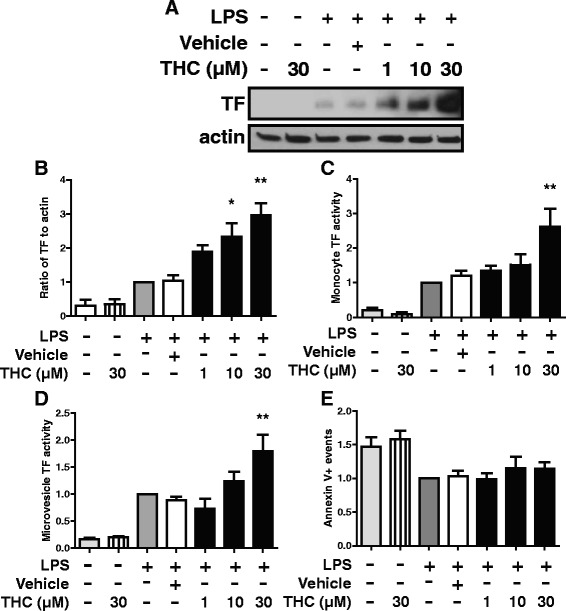
THC has dose dependent effects on TF expression and activity. Monocytes were treated with THC at indicated concentrations or vehicle control for 30 min prior to the addition of 100 ng/mL LPS for 24 h. **a** Whole cell lysates were analyzed by western blot for indicated proteins. TF western blots were stripped and reprobed for actin. **b** Densitometry showing ratio of TF to actin across 5 donors. **c** Monocyte TF activity was measured. **d**, **e** Microvesicles were prepared from supernatants from monocytes treated as in **a**-**c** and measured for **d** microvesicle TF activity or **e** counted by flow cytometry. **b**-**e** Panels represent at least 5 donors and data are expressed relative to LPS only treatment (grey bar). Graphs show mean and standard error. * *p* < 0.05, ** *p* < 0.01 via ANOVA followed by Bonferroni post test

Enhancement of monocyte TF protein levels by THC was dose dependent with maximal production at the 30 μM dose (Fig. 2a, b). Monocyte TF activity increased 250 % at the 30 μM dose (Fig. 2c). Similar to monocyte TF activity, LPS-stimulated microvesicle TF activity showed a dose dependent THC mediated elevation (Fig. 2d). Similar numbers of microvesicles were observed by flow cytometry between THC- and vehicle-treated or untreated LPS-stimulated supernatants (Fig. 2e), indicating that TF activity increased per microvesicle rather than via increased microvesicle release by THC-treated LPS-stimulated cells. Concentrations of THC used were higher than levels reported in the circulation of individuals who use marijuana, but similar to other *ex vivo* studies of THC [17–20], perhaps reflecting serum reduction of bioactivity by THC in tissue culture models [21].

**Fig. 3:**
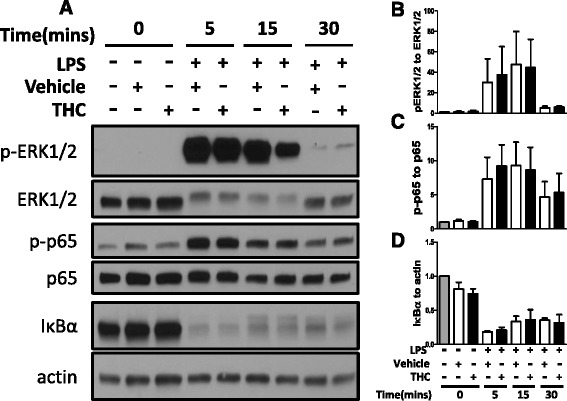
No effect of THC on selected LPS-stimulated signal transduction molecules. Monocytes were stimulated with 30 μM THC or vehicle 30 min prior to 100 ng/mL LPS for indicated time periods. **a** Whole cell lysates were subjected to western blot for indicated proteins. Representative blots of one donor are shown. Similar results were obtained in 3 donors. Phospho-ERK1/2(p-ERK1/2) blot was stripped and reprobed for total ERK1/2. Phospho-p65 (p-p65) blot was stripped and reprobed for total p65. IκBα blot was stripped and reprobed for actin. Densitometry of data from 3 donors is graphed for pERK1/2: total ERK1/2 ratio (**b**), p-p65: total p65 ratio (**c**), and IκB: actin ratio (**d**). Graphs are normalized to untreated samples and depict mean and standard error for 3 donors

**Fig. 4:**
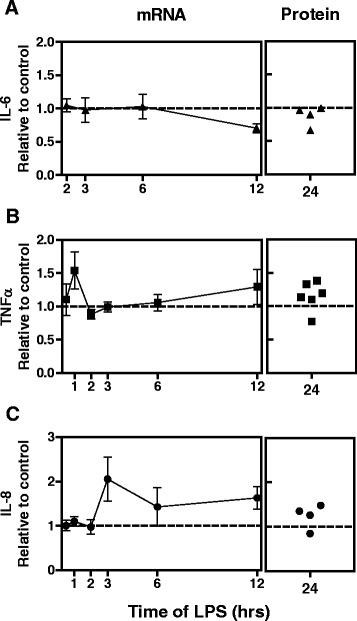
Effect of THC on pro-inflammatory cytokine stimulated by LPS. mRNA or cell supernatants (protein) from monocytes from at least 4 donors were treated with vehicle or 30 μM THC for 30 min prior to the addition of 100 ng/mL LPS for 24 h. Real-time quantitative PCR or ELISAs for (**a**) IL-6, **b** TNFα, and **c** IL-8 were performed and graphed relative to vehicle control at each time point (dotted line at 1). Left panels show mean and standard error of donors

To explore the mechanism by which THC enhanced LPS-stimulated TF expression and activity, selected signal transduction events were investigated. Although TF expression is dependent on ERK1/2 and NF-κB signaling [2, 3], phosphorylation of ERK1/2 and p65 or degradation of IκBα were unchanged (Fig. 3a-d). Since signal transduction is unaltered, it is unlikely that THC ubiquitously promotes inflammation, rather THC imparts an effect that is TF specific. Moreover, TNFα or IL-8 mRNA and secreted protein increased modestly with THC treatment, while IL-6 was unchanged (Fig. 4). In addition, while THC treatment results in modest increases in IL-8 and TNFα, THC mediated elevations in TF are of a greater magnitude. However, as TNFα stimulates TF expression [22], small elevations in TNFα expression may act synergistically with other mechanisms to further enhance THC mediated elevations in TF expression and activity.

Our results are consistent with a mechanism of THC mediated elevation of TF expression at a post-transcriptional level by inducing stabilization or preventing degradation of TF mRNA. Recently, Poly(ADP-ribose)-polymerase(PARP)-14 and tristetraprolin (TTP) were shown to cooperate to mediate TF mRNA degradation [5]. While TTP regulates mRNA transcripts of inflammatory mediators, such as TNFα [23], the addition of PARP-14 renders the complex TF specific. Since the magnitude and kinetics of TF mRNA expression (Fig. 1e) differ from TNFα (Fig. 4b), our data suggest that TTP likely plays less of a role in THC mediated elevations of TF compared to PARP-14.

Recreational marijuana use is prevalent, including among individuals with IBD and HIV-1 infection [24, 25], who are also at increased risk for coagulation disorders [8, 10]. In addition, marijuana users are likely to consume alcohol [25]. Both acute binge drinking and chronic alcohol use increase microbial translocation and circulating endotoxin [26, 27]. Taken together with our results, marijuana use alone or coupled with excessive alcohol use, may also enhance circulating procoagulant capacity.

Findings indicate that marijuana use may increase the procoagulant potential of circulating monocytes and underscore the importance of investigating the effects of marijuana use *in vivo*. Recently, several cases of sudden death in otherwise healthy individuals have linked acute marijuana use to cardiovascular complications [28]. As use of marijuana for both medicinal purposes and recreational purposes increases, investigation and close monitoring of coagulation related disorders is crucial, especially in individuals with diseases characterized by microbial translocation and dysregulated systemic inflammation.

## Abbreviations

DMEM: Dulbecco’s Modified Eagle Medium
GAPDH: Gyceraldehyde 3-phosphate dehydrogenase
IBD: Inflammatory bowel disease
IL-6: Interleukin-6
IL-8: Interleukin-8
LPS: Lipopolysaccharide
MAPK: Mitogen activated protein kinase
NF-κB: Nuclear factor κB
PVDF: Polyvinyl difluoride
SDS-PAGE: Sodium dodecyl sulfate-polyacrylamide gel electrophoresis
TF: Tissue factor
THC: Δ^9−^tetrahydrocannabinol
TNFα: Tumor necrosis factor alpha
